# An Unexpected Cause of Abdominal Discomfort: Non-pancreatic Mesenteric Pseudocyst

**DOI:** 10.7759/cureus.49459

**Published:** 2023-11-26

**Authors:** Jorge A Gutiérrez-Gónzalez, Luis A González-Torres, Gustavo Dragustinovis-Hinojosa, Néstor V Méndez-Huerta, Gerardo E Muñoz-Maldonado

**Affiliations:** 1 Department of General Surgery, Hospital Universitario Dr. José Eleuterio González, Universidad Autónoma de Nuevo León, Monterrey, MEX; 2 Department of Internal Medicine, Hospital Universitario Dr. José Eleuterio González, Universidad Autónoma de Nuevo León, Monterrey, MEX

**Keywords:** non-pancreatic pseudocyst, intra-abdominal tumors, surgical case reports, unexplained abdominal pain, mesenteric cyst, abdominal pseudocyst

## Abstract

Mesenteric cysts (MCs), rare entities of embryologic origin, predominantly affect the small bowel's mesentery. The clinical manifestations of MCs often lack specificity, which complicates diagnosis. Given their rarity, detailed reporting of MC cases is essential to enhance understanding and improve treatment strategies. We present a case of a 45-year-old male who presented to the emergency department with a one-month history of abdominal pain in the umbilical region, postprandial fullness, progressive decrease in food intake, 12 kg weight loss, and increased abdominal girth. Computed tomography (CT) imaging revealed a well-defined mass in the jejunoileal area. During the exploratory laparotomy, we identified and excised a fibrotic mass on the mesentery of the ileal jejunum, which was not adherent to the intestines. We discharged the patient with no complications following an uneventful four-day observational period. Histopathological examination, including immunohistochemical staining, confirmed the lesion as a non-pancreatic mesenteric pseudocyst. On the follow-up visit, the patient reported no complications. This case report underscores the solitary, multilocular nature of the jejunoileal MC, distinct for its serosanguineous fluid content. In conclusion, this case highlights the diagnostic challenge of MCs and illustrates the potential for successful management with a timely and multidisciplinary approach.

## Introduction

Mesenteric cysts (MCs) are a rare etiology of benign abdominal neoplasia, with an estimated incidence of one in 100,000-250,000 in different studies, the most recent published in 2002 [1-4]. Histopathological classification of MCs is crucial for tailoring treatment strategies and understanding prognostic outcomes. Ros et al. [5] delineate them into lymphangioma, mesothelial, enteric, enteric duplication, and non-pancreatic types. de Perrot et al. [6] have provided a more recent classification, dividing MCs into lymphatic, mesothelial, enteric, urogenital, dermoid, and non-pancreatic types, with similar observations compared to the previous one. This lack of consensus underscores the importance of continued research to potentially establish a unified classification. The presence of septations in non-pancreatic MCs, while rare, may indicate a more complex surgical intervention and require careful preoperative planning. Ros et al. [5] reported only one patient with septations in the non-pancreatic group, and a recent case report described another [6,7]. The seminal observations by Benevieni and the surgical advancements by Tillaux are critical historical milestones that have informed current management protocols for MCs [8]. Of the MCs presenting malignant transformation, approximately 3% had sarcomatous characteristics, primarily in the retroperitoneal space [7]. This report delves into the peculiarities of the case, such as internal septations and the jejunoileal mass' lack of adherence to the intestinal walls, facilitating a straightforward surgical resection. The successful excision of the MC via open laparotomy led to a favorable outcome, evidenced by the patient's rapid postoperative recovery and the absence of recurrent symptoms after a six-month follow-up. Cases like these emphasize the need for new research into MCs, as it is critical to advance treatment strategies and improve patient outcomes.

## Case presentation

**Figure 1:**
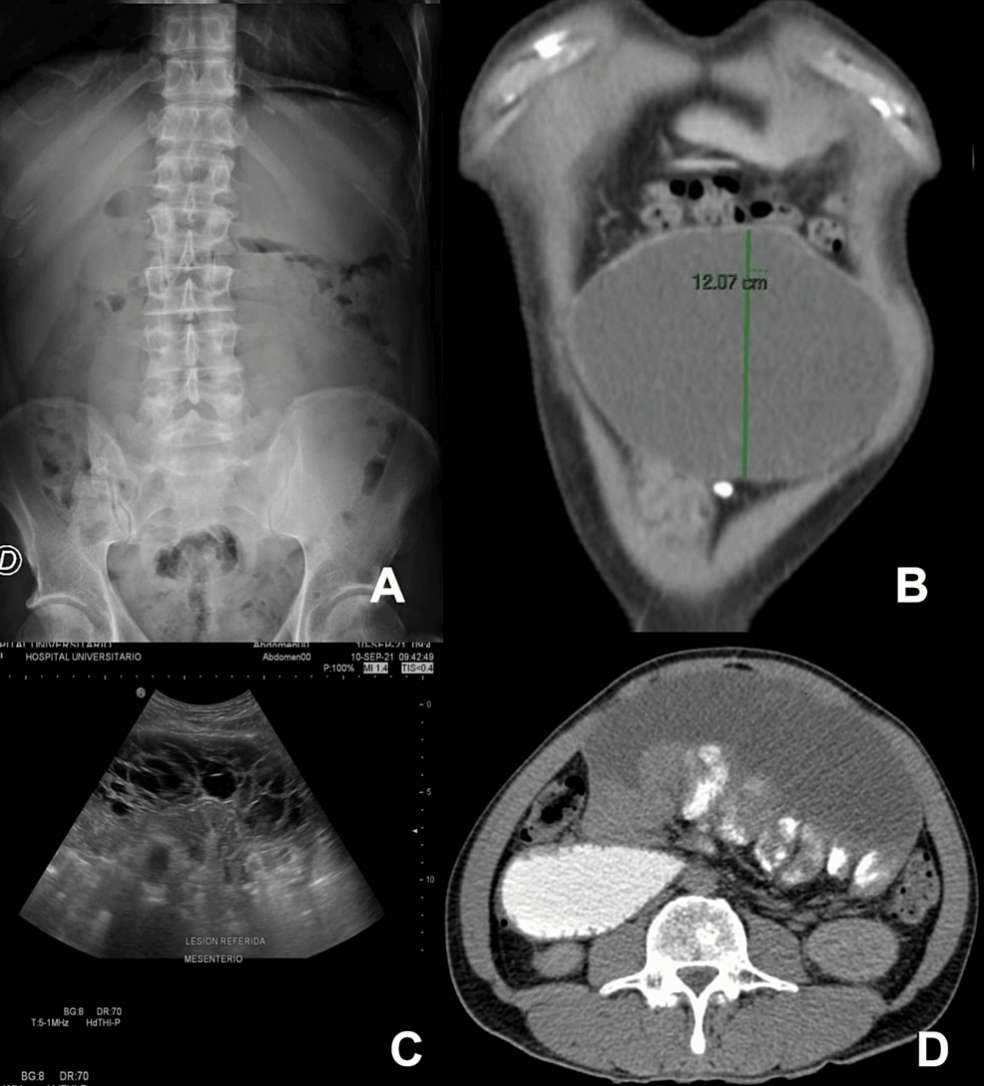
Imaging Findings of Mesenteric Mass A. Abdominal radiography reveals a non-calcified mesogastrial mass exerting pressure on abdominal structures. B. Abdominal ultrasound displaying irregular, well-defined, anechoic mesenteric lesions with multiple fine septae. C. Abdominal CT scan (coronal view) highlighting the mesenteric mass. D. Abdominal CT scan (axial view) detailing a well-defined, round, homogeneous, hypodense mass measuring approximately 15.4 × 12.07 × 7.8 cm, without contrast enhancement, equating to a volume of 756 mL. CT: computed tomography

A 45-year-old male with a past medical history that includes a surgically corrected cleft palate at age three, chronic moderate alcoholism, and denial of pancreatic or abdominal trauma presented to the emergency department with a one-month history of umbilical abdominal pain. The patient described the pain as a persistent, dull ache that worsened after meals, progressive reduction in oral intake, a 12 kg weight loss, and an increased abdominal circumference. Physical examination revealed an enlarged abdomen with a palpable, mobile, non-tender mass without signs of peritoneal irritation. Plain abdominal radiography and ultrasound imaging were pivotal in determining its mass effect on adjacent structures and ruling out other potential causes of abdominal distension, such as ascites or organomegaly (Figure 1A). Ultrasound imaging displayed an irregular, well-defined anechoic lesion with multiple fine septations in the mesentery, showing minimal Doppler flow (Figure 1B). Computed tomography (CT) imaging revealed a hypodense cystic mass without post-contrast enhancement, situated in the jejunoileal region, measuring 15.4 × 12.1 × 7.8 cm with an estimated volume of 756 mL and homogeneous content (Figures 1C, 1D), causing secondary dilatation of the duodenum and stomach. Laboratory tests indicated mild hypochloremia, which improved with isotonic crystalloid administration.

**Figure 2:**
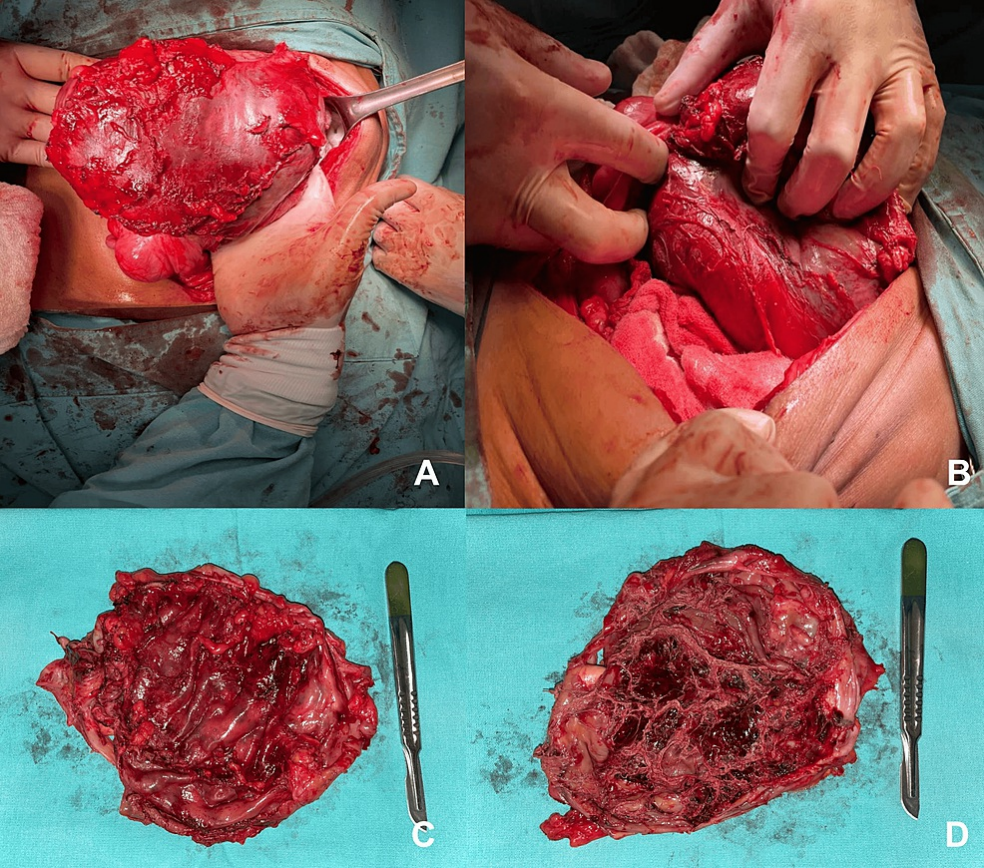
Surgical Transoperative Perspectives of the Mass A. Anterior view displaying the mass from the front. B. Lateral view illustrating the mass with a noticeable separation from the small intestine. C. External view showcasing the mass' exterior appearance. D. Internal perspective revealing the presence of internal septa within the mass.

Radiological evidence led us to suspect a benign tumor, prompting the decision to conduct an open laparotomy. This surgical approach uncovered a well-defined, fibrous mesenteric mass with few intestinal adhesions (Figure 2). Resection of the mass allowed the removal of 700 mL of serosanguineous fluid. Analysis of the fluid revealed non-significant results regarding amylase and carcinoembryonic antigen.

Postoperatively, the patient's recovery was monitored closely, with attention to hydration status and pain management, culminating in a complication-free discharge on postoperative day 4. At his two-month follow-up, the patient remained asymptomatic with no signs of recurrence on clinical examination, affirming the success of the surgical intervention. Pathological analysis of the excised tissue confirmed the diagnosis of a non-pancreatic mesenteric pseudocyst discarding malignancy.

## Discussion

The main strengths of this case report are the clinical and radiological findings, which furnish critical insights for both generalists and specialists. Notably, the conclusions drawn from these findings could be more accurate when juxtaposed with extant literature on mesenteric cysts (MCs). However, a primary limitation of this study is the absence of histopathological imaging evidence, attributable to restrictions about hospital-patient confidentiality that precluded its procurement.

As the introduction highlights, mesenteric cysts (MCs) are infrequent clinical findings. Regarding their histopathological features, Perrot et al. [6] described the most recent classification system. The etiological factors contributing to mesenteric cysts (MCs) development remain unclear.

Since 1985, five significant case series have elucidated aspects of MCs. Kurtz et al. [3] conducted a comprehensive descriptive analysis, synthesizing data from 10 of their cases along with 152 from existing literature. Among these, 98 patients were at least a decade old. Their findings indicated a predominance of MCs in females and abdominal pain as the most frequent presenting symptom. The small intestine emerged as the most affected site, implicated in 51% of cases. Additionally, they noted a 3% incidence of malignancy. Tan et al. [2] showed 16 MC cases where they reported near equal gender presentation, abdominal pain, and mass were the most prevalent symptoms/signs. More than 50% of the patients required a CT scan to make the MC diagnosis; the most standard site of appearance was the retroperitoneum, and laparoscopic surgical resection occurred in 75% of patients. Santana et al. [9] documented 18 MC cases, noting a female predominance at 72.2%. Abdominal pain was the chief complaint, with mesothelioma and lymphangioma being the most common types, each accounting for 44.4% of cases. The fluid content was predominantly serous in 33.3% of cases and serosanguineous in 11%; three patients presented with septal imaging findings. Prakash et al. [10] examined 17 pediatric cases of MC. In the latest study by Yavuz et al. [1] from 2021, a series involving 22 patients with mesenteric cysts demonstrated a female predominance. The predominant clinical symptom reported was abdominal pain, and a significant majority of cases (81%) exhibited involvement of the small intestine. Table 1 provides a summary of the findings from these publications.

**Table 1:** Relevant MC Evidence The table chronologically presents relevant evidence about MCs and highlights key findings. MC: mesenteric cyst, CT: computed tomography

Publication year	Author	Relevant findings
1986	Kurtz et al. [3]	Case series of 162 patients with mesenteric cysts, 62% were female, and 38% were male. Of these, 98 patients were older than 10, while 64 were younger than 10. Abdominal pain emerged as the most common symptom. Regarding location, 60% of cysts developed in the small intestine and 24% in the large. Most patients underwent concurrent intestinal resection. Five patients developed malignancy, and a recurrence of 11% occurred.
2009	Tan et al. [2]	Case series of 16 patients with mesenteric cysts, females comprised 56.2% and males 43.8%. Abdominal pain affected 62.5% of the patients, 43.8% had an abdominal mass, and 6.3% experienced acute intestinal obstruction. A CT scan was necessary to diagnose 75% of the cases. The cyst locations were as follows: 31.3% in the retroperitoneum, 25% in the sigmoid mesocolon, and 25% in the small bowel. Surgeons performed laparoscopic resections in 75% of the cases and laparotomies in the remaining 25%, with no recurrences reported.
2010	Santana et al. [9]	Case series of 18 patients with mesenteric cysts, 72.2% were female, and 27.8% were male. The most common symptoms included abdominal pain and the presence of a mass. Physicians performed CT scans for accurate diagnoses. The types of cysts were lymphangioma and mesothelioma, each constituting 44.5% of cases, followed by hemorrhagic and mucin cysts at 5.5% each; only three cases presented multiple septae. The fluid content was serous in 33.3% of cases, serosanguineous in 11.1%, mucoid in 16.7%, chylous in 5.5%, and serosanguineous chylous in 5.5%, with 27.8% unclassified.
2010	Prakash et al. [10]	In this pediatric study group, all 17 patients experienced abdominal pain and mass as the most frequent symptoms. Each patient had a diagnosis of lymphangiomatous mesenteric cysts.
2021	Yavuz et al. [1]	Case series of 22 patients, 63.6% were female, and 36.4% were male. Abdominal pain emerged as the most frequent symptom among them. Doctors used CT scans to diagnose all patients. The small intestine was involved in 81.8% of cases and the colon in 18.3%. The surgical approaches varied: 50% underwent enucleation, 18.2% had small bowel resection and anastomosis, 13.6% received laparoscopic cyst excision, and 4.5% each had a left or right hemicolectomy. Seventeen patients had simple cysts, four had lymphangioma, and one had a simple cyst concurrent with colon cancer. Postsurgical complications included a 13.6% rate of surgical site infection, and 4.5% experienced anastomosis leakage.

Our case exhibits several characteristics that align with those reported in the literature. As highlighted previously, abdominal pain is the most observed symptom across all studies; the CT scan remains the most valuable diagnostic tool for preoperative evaluation. The reported tumor sizes range from 2 to 27 cm [1], and our case falls within this spectrum with a tumor measuring 14 cm. Like most reported instances, our case involved the small intestine. Although septations are rare, they were present in our patient. The characteristic of serosanguineous fluid is a rare finding, adding a unique aspect to our case.

Adhesions are frequently observed in up to 50% of patients, often necessitating intestinal resection with careful dissection of adjacent structures for complete removal [7]. In contrast, our case exhibited few adhesions, facilitating a less complex and more straightforward total resection.

Aspiration and marsupialization are generally discouraged due to the potential for increased complications [2]. Conversely, enucleation seems promising, with a low recurrence rate (0%-13.6%) and an excellent prognosis, particularly in pediatric cases [10]. Treatment choices may differ based on institutional practices and the surgical team's preoperative assessment. We chose to perform an open laparotomy due to the considerable size of the tumor mass.

Reported complications following surgical intervention for mesenteric cysts include surgical site infections, with an incidence of 13.6%, and anastomotic leaks, occurring in 4.5% of cases involving resection with anastomosis [7].

## Conclusions

Our case report shares features commonly noted in the literature but stands out due to some rare findings. The symptoms of abdominal pain, the size of the cyst, and the diagnostic methods used align with previous reports. However, septations, the specific histological subtype, and the preservation of organ integrity during treatment underscore its uniqueness, with noteworthy clinical and surgical implications. Despite opting for a less conventional treatment route, we encountered no complications.

Highlighting mesenteric cysts (MCs) in medical literature is crucial due to their rare incidence. The predominance of low-level evidence in the form of case reports and series underscores the need for a comprehensive analysis of the data. Current classification systems need to be more consistent, and the lack of uniform reporting of histopathological features in the literature indicates an urgent need for consensus on reporting standards for such cases.

## References

[1] Yavuz Y, Varman A, Şentürk ÜM, Kafadar MT (2021). Mesenteric cyst in 22 cases. J Gastrointest Cancer.

[2] Tan JJ, Tan KK, Chew SP (2009). Mesenteric cysts: an institution experience over 14 years and review of literature. World J Surg.

[3] Kurtz RJ, Heimann TM, Holt J, Beck AR (1986). Mesenteric and retroperitoneal cysts. Ann Surg.

[4] Huis M, Balija M, Lez C, Szerda F, Stulhofer M (2002). [Mesenteric cysts]. Acta Med Croatica.

[5] Ros PR, Olmsted WW, Moser RP Jr, Dachman AH, Hjermstad BH, Sobin LH (1987). Mesenteric and omental cysts: histologic classification with imaging correlation. Radiology.

[6] de Perrot M, Bründler M, Tötsch M, Mentha G, Morel P (2000). Mesenteric cysts. Toward less confusion?. Dig Surg.

[7] Ayas MF, Affas S, Hoilat GJ, Kassab I, Barawi M (2020). A case of a mesenteric cyst mimicking a biloma. Gastroenterology Res.

[8] Carvalho NM, Lopes Filho JA, Plens IC, Camara VA, Teixeira CC, Figueiredo PH, Araujo Junior ON (2020). Mesenteric cyst presenting with acute abdomen pain and bowel obstruction: case report and brief literature review. Ann Med Surg (Lond).

[9] Santana WB, Poderoso WL, Alves JA, Melo VA, Barros Cd, Fakhouri R (2010). [Mesenteric cyst--clinical and pathological aspects]. Rev Col Bras Cir.

[10] Prakash A, Agrawal A, Gupta RK, Sanghvi B, Parelkar S (2010). Early management of mesenteric cyst prevents catastrophes: a single centre analysis of 17 cases. Afr J Paediatr Surg.

